# Circular RNA hsa_circ_001783 regulates breast cancer progression via sponging miR-200c-3p

**DOI:** 10.1038/s41419-018-1287-1

**Published:** 2019-01-22

**Authors:** Zihao Liu, You Zhou, Gehao Liang, Yun Ling, Weige Tan, Luyuan Tan, Robert Andrews, Wenjing Zhong, Xuanxuan Zhang, Erwei Song, Chang Gong

**Affiliations:** 1 Breast Tumor Center, Guangdong Provincial Key Laboratory of Malignant Tumor Epigenetics and Gene Regulation, Sun Yat-sen Memorial Hospital, Sun Yat-sen University, Guangzhou, China; 2 Systems Immunity University Research Institute and Division of Infection and Immunity, School of Medicine, Cardiff University, Cardiff, UK; 3 Department of Breast Surgery, First Affiliated Hospital of Guangzhou Medical University, Guangzhou, China; 4 Fountain-Valley Institute for Life Sciences, 4th Floor, Building D, Guangzhou Institute of Biomedicine and Health, Chinese Academy of Sciences, 190 Kaiyuan Avenue, Huangpu District, Guangzhou, China

## Abstract

Increasing evidence suggests circular RNAs (circRNAs) exert critical functions in tumor progression via sponging miRNAs (microRNAs). However, the role of circRNAs in breast cancer remains unclear. Here we systematically analyzed the circular RNAs in breast cancer based on their characteristic in sponging disease-specific miRNAs and identified hsa_circ_001783 as a top ranked circRNA in our computation and verified its high expression in both breast cancer cells and cancer tissue. A higher level of hsa_circ_001783 was significantly correlated with heavier tumor burden and poorer prognosis of patients with breast cancer. Knockdown of this circRNA remarkably inhibited the proliferation and invasion of breast cancer cells. Importantly, hsa_circ_001783 promoted progression of breast cancer cells via sponging miR-200c-3p. Taken together, hsa_circ_001783 may serve as a novel prognostic and therapeutic target for breast cancer.

## Introduction

Breast cancer is the most frequently diagnosed cancer for women in the world^1,2^. Despite recent advances in early diagnosis and effective treatment, breast cancer in some patients would progress to metastatic stage after therapy without knowing the reason. Therefore, it is essential to search for novel molecules in order to understand the progression of breast cancer.

Circular RNAs (circRNAs) were first detected in virus as covalently closed looped RNAs^3^. As next-generation sequencing technologies are developing rapidly, a number of circRNAs have been identified as functional molecules in regulating disease progression rather than splicing by-products^4–6^. Our previous study has demonstrated that circRNAs can promote breast cancer cells progression under hypoxia^7^. Others have revealed circRNAs contribute to breast cancer proliferation and invasion^7–9^. Further studies indicate that imperfect matches could be formed in circRNA-miRNA duplex, which enable circRNAs to serve as “miRNA sponge” and prevent miRNA-mediated degradation of mRNAs^10^. For example, CDR1as sponges miR-7 via its miR-7 targeting sites and regulates tumor progression^11,12^. CircHIPK3, circGFRA1, and hsa_circ_0001982 have been reported as functional miRNA sponges in cancers^8,9,13^. These studies focused on the differentially expressed circRNAs rather than elucidating their sponge ability and the role of circRNAs in breast cancer remains obscure. Thus, there is an urgent need to characterize their sponge abilities and define the associated molecular mechanism in breast cancer.

In the present study, we proposed a new bioinformatics method to screen “circular sponges”. We used five algorithms to predict binding sites of human miRNAs to the conserved sequences of individual circRNAs. Simultaneously, we identified breast cancer-associated miRNAs using Ingenuity knowledge database, Pubmed, and Embase. Five essential functional features were used to score the strength associations between miRNAs and breast cancer. And the network branches across circRNA, miRNA, and breast cancer were ranked. We further assess the clinical potential and explore the molecular function of the top ranked circRNA in breast cancer.

## Material and methods

### Data extraction and analysis

CircRNA annotations and sequences were extracted from circBase^14^. MiRNA sequences were extracted from miRBase^15^. The conserved circRNA sequences were analyzed as described^16^. Five algorithms including Targetscan^17^, miRanda^18^, PITA^19^, RNAhybrid^20^, and RNA22 (ref. ^21^) were used to analyze the potential bindings of miRNAs to individual circRNA. The potential targets of individual miRNAs were predicted by starbase with summation of targetScan sites, picTar sites, RNA22 sites, PITA sites, and miRanda sites ≥5 (ref. ^22^). Two miRNA microarray datasets (GSE40056 and GSE28969) and one mRNA microarray dataset (GSE41313) were downloaded from NCBI GEO public database (www.ncbi.nlm.nih.gov/geo) and analyzed by R version 3.4.3. The log2FC > 1.5 and *P* < 0.05 were characterized as differentially expressed miRNAs or mRNAs. The circRNA–miRNA–mRNA was visualized by Cytoscape (version 3.6.0). The miRNA gene ontology (GO) terms “biological process” analysis was constructed by Cytoscape plug-in ClueGo^23^. The mRNA GO terms were analyzed by DAVID (https://david.ncifcrf.gov/).

### Patient samples and clinical database

A total of 136 breast cancer patients aged from 18 to 70 years old enrolled Sun Yat-sen Memorial Hospital (SYSMH) between 1 June 2010 and 31 May 2015. Their paraffin-embedded tissue samples and paired non-tumorigenesis tissue samples (*n* = 18) were collected for RNA fluorescence in situ hybridization (FISH). Patients who received neoadjuvant chemotherapy were excluded. All the HER2 (human epidermal growth factor receptor 2)-positive patients received anti-HER2 therapy. In addition, a total of 50 fresh-frozen cancer specimens were collected from breast cancer patients who received no therapy before surgery at the Breast Tumor Center of SYSMH. The specimens were stored in RNA later (Ambion, USA) at −80 °C immediately and were used for qPCR analysis afterwards.

### RNA FISH

Cy3-labeled oligonucleotide probe for hsa_circ_001783 and FAM-labeled oligonucleotide probe for hsa-miR-200c-3p were applied for RNA FISH. The oligonucleotide sequences are available in the Supplementary Information. Paraffin section of breast cancer samples were deparaffinized with 100% xylene and rehydrated with different graded ethanol. For RNA FISH of co-localization of hsa_circ_001783 and hsa-miR-200c-3p, cells were seeded in a glass-bottom dish. Then they were incubated with prehybridization solution at 37 °C for 30 min and the probes (Ribobio, China, 20 μM) were added to slides or dish individually and hybridized overnight. Then they were washed with buffer I (4× SSC, 0.1% Tween-20) for three times, wash with buffer II (2 × SSC) for once, and wash with buffer III (1× SSC) for once. After being washed with phosphate-buffered saline, they were incubated with DAPI to stain cell nuclear. The cells at each staining intensity were recorded on a scale of 0 (no staining), 1 (light red), 2 (red), 3 (strong red), and 4 (dark red). The staining index (SI) was calculated as follows: SI = staining intensity × proportion of positively stained cells. Positive cells in the whole fields of view were calculated.

### RNA-binding protein immunoprecipitation (RIP)

The RIP assay was performed by Magna RIP Kit (Millipore, USA) and was conducted as previously instructed^13^. Briefly, 1 × 10^7^ cells of HEK-293T were incubated with lysis buffer with protease and RNase inhibitors added. Then the cell lysis was incubated with magnetic beads which are conjugated with human ani-Argonaute2 (AGO2) antibody (Millipore, USA) or negative control IgG (Millipore, USA), respectively, at 4 °C overnight. Subsequently, samples were washed and incubated with Proteinase K. Immunoprecipitated RNA was purified and was subjected to quantitative real-time PCR analysis to determine hsa_circ_001783.

### CircRNA pull-down

Biotin-labeled hsa_circ_001783 probe and control probe (Sangon Biotech, China) were used for circRNA pull-down and the assay was performed as mentioned previously^24–26^. In brief, MDA-MB-468 was cross-linked by 1% formaldehyde for 30 min, lysed in co-IP buffer, and centrifugated. The supernatant was incubated with hsa_circ_001783-specific probes-streptavidin beads (Life Technologies, USA) mixture overnight at 37 °C. On the next day, the samples were washed and incubated with lysis buffer and proteinase K. Finally, the mixture was added with TRIzol reagent for RNA extraction and followed by detection of hsa_circ_001783, hsa-miR-200c-3p, and β-actin.

### AGO-binding sites from PAR-CLIP data sets

The AGO1 and AGO2 binding sites were acquired from published photoactivatable cross-linking immunoprecipitation (PAR-CLIP) data in GEO database (https://www.ncbi.nlm.nih.gov/geo/). Three PAR-CLIP datasets (GSE28865, GSE43573, and GSE21918) from HEK293 cells were extracted. We analyzed the AGO1 as well as AGO2 binding sites of hsa_circ_001783 genomic region.

### Cell lines and treatment

The human non-carcinogenesis mammary epithelial cell line (MCF-10A) and human breast tumor cell lines T47D, BT474, SK-BR-3, MCF-7, MAD-MB-468, and MDA-MB-231 were obtained from American Type Culture Collection (ATCC). All cell lines which were passaged less than 6 months were authenticated by short tandem repeat DNA profiling within 6 months and were cultured according to the recommended protocols. MDA-MB-231 and MDA-MB-468 were transfected with small interfering RNA and miR-200c-3p inhibitor (GenePharma, China) using Lipofectamine^TM^ 3000 (Invitrogen, MA, USA).

### Luciferase report assay

The conserved sequences of hsa_circ_001783 were cloned into pGL3-enhancer vector between *BgI*II and *Sam*I sites. 3 × 10^4^ cells of MDA-MB-231 and MDA-MB-468 were seeded in a 24-well plate individually and co-transfected with 300 ng pGL3-has_circ_001783 as well as pGL3 control vector. Subsequently, cells were transfected with mimics negative control, inhibitor control, hsa-miR-200c-3p mimics, or inhibitor respectively. After 24 h of transfection, the luciferase assay was conducted using dual luciferase reporter assay (Vazyme, China) according to the manufacturer’s instructions.

### Statistical analysis

Pearson chi-square test or Fisher^’^s exact test was performed for categorical values. Mann–Whitney *U* test and Kruskal–Wallis test were used to determine the differences between groups. Mann–Whitney *U* test was applied to evaluate the association between has_circ_001783 levels and various clinical pathological variables in breast cancer patients. Pearson’s correlation coefficient analysis was used to assess the linear correlations. Survival rates and curves were determined by the Kaplan–Meier method, and the comparison of survival differences was evaluated by using the log-rank test. COX regression analysis was used for univariate and multivariate analysis of correlation between clinical pathological variables and survival. All data statistical analyses were performed using Graphpad Prism version 6.0 (GraphPad Software Inc., San Diego, CA, USA) and SPSS version 20.0 (SPSS Inc., Chicago, IL, USA). In all cases, *P* values less than 0.05 were considered statistically significant. All statistical tests were two-sided.

### Additional experiment procedures

Colony formation assay, migration and invasion assay, immunohistochemistry, CCK8 assay, EdU assay, nuclear–cytoplasmic fraction assay are provided in Supplementary Information.

## Results

### Identification and characterization of hsa_circ_001783 via circRNA–miRNA–breast cancer network

**Fig. 1:**
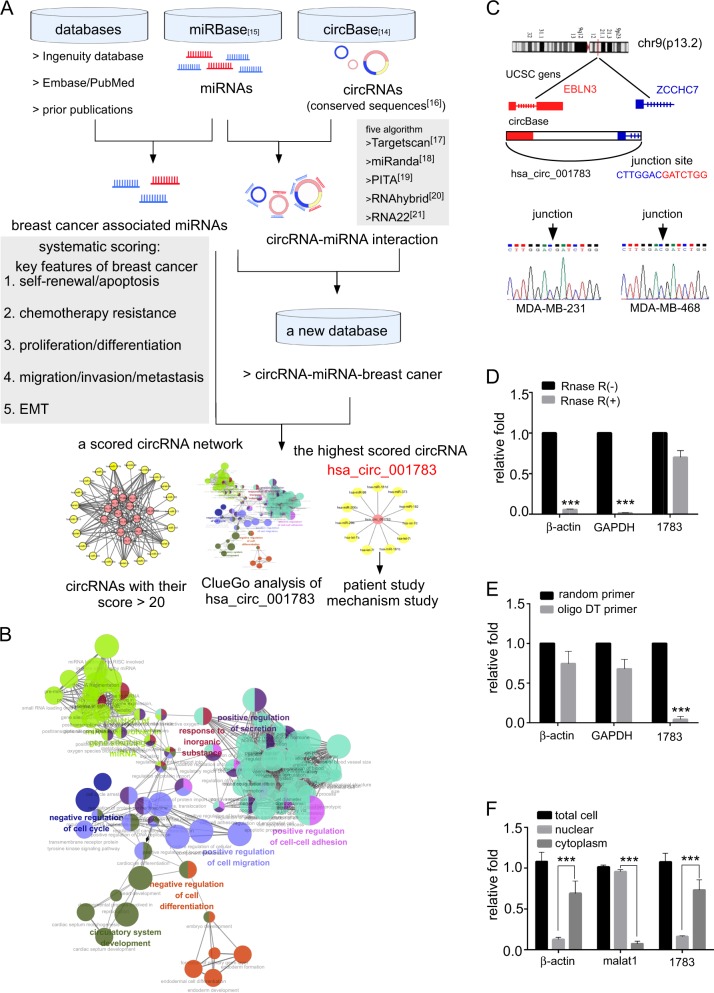
Screening circRNA candidates in breast cancer and characteristics of hsa_circ_001783. **a** Workflow of screening circRNA candidates in breast cancer. **b** The biological process ontology terms of hsa_circ_001783. **c** The genomic loci of hsa_circ_001783 and the Sanger sequence of junction site of hsa_circ_001783 in two breast cancer cell lines: MDA-MB-231 and MDA-MB-468. **d** qPCR analysis of β-actin, GAPDH, and hsa_circ_001783 (1783) after Rnase R treatment. ***P < 0.001 compared to no treatment group. **e** qPCR analysis of poly A-tailed mRNAs including β-actin and GAPDH which can be synthesized to cDNA by using random primer or oligo DT primer alone and non-poly A-tailed RNA hsa_circ_001783 (1783) which cannot be reversed to cDNA by using oligo DT primer. ***P < 0.001 compared to the random primer. **f** qPCR analysis of hsa_circ_001783 in the cytoplasm and nuclear of which was separated by PARIS kit. β-Actin mRNA in cytoplasm and long non-coding RNA malat1 residing in nuclear fraction were referred as quality controls of nuclear and cytoplasm fractions, respectively. Total RNA was total portion of the nuclear plus cytoplasm and was referred as the control. ***P < 0.001 compared to the cytoplasm

We performed our analysis according to the procedure shown in Fig. 1a. Five algorithms, Targetscan, miRanda, PITA, RNAhybrid, and RNA22 were used to predict the potential bindings of miRNAs to the conserved sequences of individual circRNAs (Supplementary Table 1). We identified 923 circRNAs binding to 100 miRNAs through more than 37,000 potential interactions. Screening ingenuity knowledge base, PubMed, and Embase databases enables us to find breast cancer-associated miRNAs. After merging the data together, we identified 594 breast cancer associated-circRNAs. Based on our prior knowledge, five essential features including self-renewal/apoptosis, chemotherapy resistance, differentiation/proliferation, migration/invasion/metastasis, and epithelia–mesenchymal transition (EMT) of breast cancer cells were employed to rank the circRNAs across the newly constructed circRNA–miRNA–breast cancer database (Supplementary Table 2). We found hsa_circ_001783 had the highest score among other 594 circRNAs (Fig. 1a; Supplementary Table 2). The ClueGo analysis of its targeted miRNAs revealed hsa_circ_001783 was involved in enriched GO biological processes such as cancer metastasis (e.g. cell migration and cell–cell adhesion) and proliferation (e.g. regulation of cell cycle and cell differentiation) (Fig. 1b).

### Characterization of molecular structure of hsa_circ_001783

Apart from intergenic region, hsa_circ_001783 (chr9: 37086664–37121124) is partially derived from exon 2 in human endogenous Bornavirus-like nucleoprotein 3 (EBLN3), and exon 1 as well as intron 1 in zinc-finger CCHC-type containing 7 (ZCCHC7; Fig. 1c). The genomic sequence of hsa_circ_001783 is 34460nt. To confirm the circular characteristics of hsa_circ_001783, we next digested total RNA with or without Rnase R which has a 3′-5′exoribonuclease activity^27^. Compared to the linear β-actin or GAPDH mRNAs, hsa_circ_001783 was obviously resistant to Rnase R (Fig. 1d). Since circRNAs generally lack poly A tail due to its covalently closed looped structure, we then used random primer or oligo DT primer alone to synthesize cDNA. Poly A-tailed mRNAs, β-actin, and GAPDH could be reversed to cDNA by either random primer or oligo DT primer, while hsa_circ_001783 could only be reversed by random primer only (Fig. 1e). These suggest that hsa_circ_001783 possesses a loop structure. In addition, we found 80% of hsa_circ_001783 located in cytoplasm (Fig. 1f).

### High level of hsa_circ_001783 is correlated with poor clinical outcomes in breast cancer patients

**Fig. 2:**
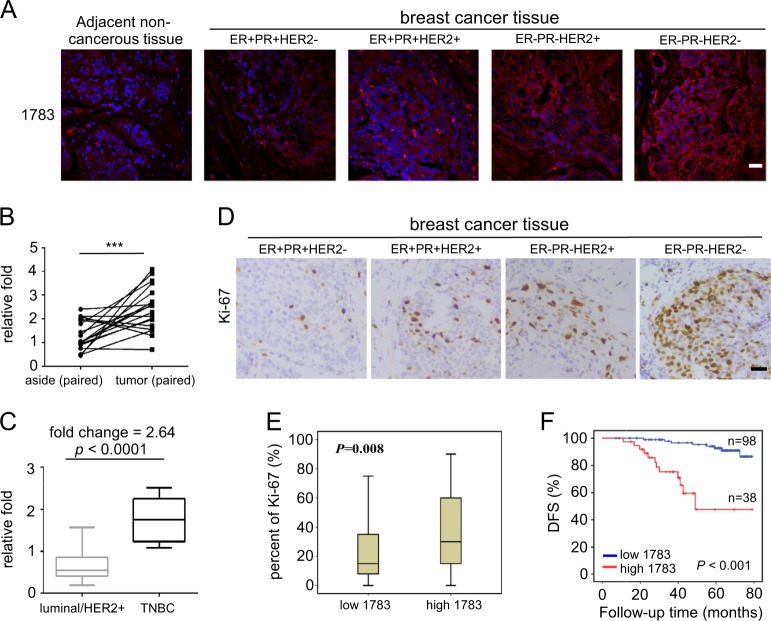
Hsa_circ_001783 is up-regulated and correlated with poor clinical outcomes in breast cancer patients. **a** The cellular location and relative expression of hsa_circ_001783 in breast cancer tissue and adjacent non-cancerous tissue. The nuclear was stained with DAPI for blue color and hsa_circ_001783 was stained for red color. Scale bar, 20 μm. **b** The level of hsa_circ_001783 in breast cancer tissue compared to paired non-cancerous tissue (*n* = 18) analyzed by FISH. ***P < 0.001 compared to the level of hsa_circ_001783 in paired breast cancer tissue. **c** The expression of hsa_circ_001783 in frozen fresh breast cancer samples analyzed by qPCR (TNBC *n* = 11, Luminal/HER2+ *n* = 39). **d** The corresponding Ki-67 staining of breast cancer samples which has been used for FISH analysis. Scale bar, 20 μm. **e** The box plot of Ki-67 index in high and low hsa_circ_001783 subgroups. *P* = 0.009 compared to the Ki-67 in low hsa_circ_001783. Chi-square test was performed to analyze correlation between hsa_circ_001783 and Ki-67. *X*^2^ = 7.119, *P* = 0.008. **f** The disease-free survival (DFS) curves of 136 breast cancer patients with high or low hsa_circ_001783 expression. *ER* estrogen receptor, PR progesterone receptor, HER2 human epidermal growth factor receptor 2

We further assessed the association between hsa_circ_001783 expression and pathological characteristics. Primary tumors from 136 breast cancer patients were divided into two groups based on the mean expression of hsa_circ_001783. We found that hsa_circ_001783 expression was significantly correlated with tumor size (*P* < 0.001), lymph node (LN) status (*P* *<* 0.001), TNM stage (*P* *<* 0.001), ER status (*P* *=* 0.02), PR status (*P* *<* 0.001), molecular subtype (TNBC vs. non-TNBC, *P* *<* 0.001), and Ki-67 index (*P* *=* 0.008), but not with age, menopause, HER2 status, and histological grade (Table 1). In addition, RNA FISH showed that hsa_circ_001783 was remarkably over-expressed in breast cancer tissue compared to paired non-cancerous tissue (Fig. 2a, b). Furthermore, this circRNA was upregulated in ER-PR-HER2- (triple negative, TN) subtype compared to the luminal and HER2 amplification (HER2+) subtypes (Fig. 2a). Being consistent with FISH results, qPCR showed that hsa_circ_001783 was upregulated by 2.64-fold high (*P* < 0.0001) in tumor samples of triple-negative breast cancer (TNBC, Fig. 2c). Since hsa_circ_001783 was upregulated in tumors with higher proliferation potential, we further examined the relationship between the hsa_circ_001783 expression and Ki-67 proliferation index. We found that hsa_circ_001783 was positively correlated with the Ki-67 level in breast tumors (*P* = 0.009; Fig. 2d, e). Our Kaplan–Meier analysis further revealed that patients with higher level of hsa_circ_001783 were more likely to develop disease recurrence and had poor disease-free survival (*P* *<* 0.001; Fig. 2f). Multivariate analysis showed that the expression level of hsa_circ_001783 was an independent factor for predicting the prognosis of breast cancer patients (hazard ratio, HR: 9.114; 95% confidence interval, 95% CI: 2.428–34.206, *P* *=* 0.001; Supplementary Table 3).

**Table 1:** Clinical pathological variables of breast cancer patients

Variables	Classifier	Low Hsa_circ_001783	High Hsa_circ_001783	Total	*P* value	RR
		*n* = 98	*n* = 38	*n* = 136		
Age	≤40 years	16	7	23	0.77	0.864
	>40 years	82	31	113		
Menopause	Yes	63	27	90	0.454	0.733
	No	35	11	46		
Tumor size	≤2 cm	38	3	41	<0.001	7.389
	>2 cm	60	35	95		
LN status	Negative	53	3	56	<0.001	13.741
	Positive	45	35	80		
TNM stage	I	31	1	32	<0.001	17.119
	II–III	67	37	104		
Tumor grade	I	9	2	11	0.727	1.82
	II–III	89	36	125		
ER	Negative	22	19	41	0.002	0.289
	Positive	76	19	95		
PR	Negative	19	19	38	<0.001	0.241
	Positive	79	19	98		
HER2	Negative	82	35	117	0.203	0.439
	Positive	16	3	19		
Molecular subtype	TNBC	10	21	31	<0.001	0.092
	Non-TNBC	88	17	105		
Ki-67	≤14%	45	8	53	0.008	3.184
	*>* *14%*	*53*	*30*	*83*		

*LN* lymph node, *ER* estrogen receptor, *PR* progesterone receptor, *HER2* human epidermal growth factor receptor 2, *RR* relative risk, *TNBC* triple-negative breast cancer

### Construction of co-expression network, GO, and KEGG (Kyoto Encyclopedia of Genes and Genomes) pathway analysis

**Fig. 3:**
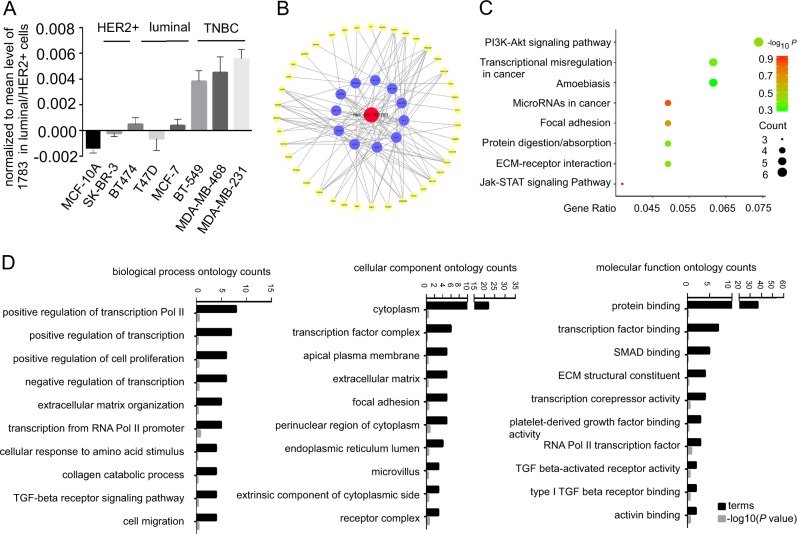
KEGG pathway analysis and gene ontology of hsa_circ_001783 co-expressed genes. **a** The basal level of hsa_circ_001783 in breast cancer cells of different subtypes. **b** Constructed hsa_circ_001783–miRNA–mRNA network with a mRNA positive correlation co-efficiency cut-off value >0.1. Red ellipse: hsa_circ_001783; blue ellipse: miRNAs potentially interacted with hsa_circ_001783; yellow ellipse: genes positively co-expressed with hsa_circ_001783. **c** KEGG pathway analysis of hsa_circ_001783-co-expressed genes. Count represents the number of genes enriched in the cluster; gene ratio is the proportion of each count in the total mRNAs of the hsa_circ_001783–miRNA–mRNA network. **d** The cellular component ontology, molecular function ontology, and biological process ontology analysis of hsa_circ_001783-co-expressed genes. Terms: gene ontology annotation terms; ontology counts: the number of genes enriched in the cluster

We quantified the expression levels of hsa_circ_001783 in different breast cancer cell lines, analyzed the genes correlated with hsa_circ_001783 expression, and performed GO analysis to predict the potential functions. The basal level of hsa_circ_001783 was much higher in breast cancer cell lines than non-tumorigenesis breast mammary cell line MCF-10A (Fig. 3a). Consistent with the level of hsa_circ_001783 in breast cancer samples, we found that hsa_circ_001783 was 2.5–3-folds higher in TNBC cell lines (MDA-MB-231, MDA-MB-468, and BT-549) than luminal and HER2-overexpression cell line (BT-474, MCF-7, T47D, and SK-BR-3) (Fig. 3a). To further predict the potential function of hsa_circ_001783, we analyzed the positively co-expressed genes of hsa_circ_001783 in MDA-MB-231, MDA-MB-468, and BT-549 with the Pearson correlation co-efficiency cut-off value >0.1 (GSE41313; Supplementary Tables 3 and 4). Based on the binding sites of miRNAs in the positively co-expressed genes and the hsa_circ_001783 predicted targeting miRNAs, we constructed a hsa_circ_001783–miRNA–mRNA network (Fig. 3b). We then employed KEGG pathway and GO to analyze the biological functions of genes in this hsa_circ_001783–miRNA–mRNA network, assuming that the hsa_circ_001783 may have molecular interactions with these genes, or could be involved in regulating biological functions of these genes. Hsa_circ_001783-correlated genes were enriched in cancer-associated KEGG pathways including PI3K–Akt signaling pathway, transcriptional misregulation in cancer, microRNA in cancer, and focal adhesion (Fig. 3c). Hsa_circ_001783 were significantly correlated with genes in the biological process of genes ontology, including *IL6*, *ETS1*, *E2F7*, *SIX1*, *ZEB1*, *COL4A1*, *COL3A1*, and *COL1A1,* which were enriched in regulation of transcription, regulation of cell proliferation, collagen catabolic process, and cell migration. In the analysis of cell component ontology, hsa_circ_001783-correlated genes, such as *IL6*, *LMO7*, *ZEB1*, *ETS1,* *SIX1* and *MSN*, were mainly enriched in cytoplasm, transcription factor complex, extracellular matrix, and focal adhesion, respectively. In the ontology analysis of molecular function, hsa_circ_001783-correlated genes (including *E2F7*, *COL3A1*, *ZEB1*, *MSN*, *RUNX2*, *IL6*, *COL4A2*, *COL4A1*, *TGFBR2*, *CLIC4*, *ETS1*, *SIX1*, *CYBRD1*, *TGFBR3*, *MAPRE2* and *COL1A1*) were enriched in protein binding, transcriptional factor binding, SMAD binding, and ECM structural constituent, respectively (Fig. 3d). Taken together, the KEGG and GO analysis suggest that hsa_circ_001783-correlated genes are associated with cancer growth and metastasis (Table 2).

**Table 2:** Multivariate Cox analysis of variables considered for disease-free survival rates of breast cancer patients

Variables	Category	Multivariate			
		HR	95% CI lower	95% CI upper	*P* value
Age	>40 vs. ≤40 years	0.645	0.218	1.91	0.429
Tumor size	>2 vs. ≤2 cm	1.743	0.402	7.548	0.458
LN status	Positive vs. Negative	3.435	0.723	16.324	0.121
TNM stage	Stage II–III vs. I	0.387	0.065	2.313	0.298
Grade	Grade II–III vs. I	0.105	0.018	0.613	0.012
ER status	Positive vs. Negative	0.767	0.141	4.182	0.759
PR status	Positive vs. Negative	0.258	0.044	1.528	0.135
HER2 status	Positive vs. Negative	1.451	0.203	10.381	0.711
Ki-67 status	>14% vs. ≤14%	3.418	0.823	14.193	0.091
Molecular subtype	TNBC vs. non-TNBC	4.459	0.48	41.439	0.189
Hsa_circ_001783	High vs. ≤Low	9.114	2.428	34.206	0.001

*LN* lymph node, *ER* estrogen receptor, *PR* progesterone receptor, *HER2* human epidermal growth factor receptor 2, *HR* hazard ratio, *CI* confidence interval, *TNBC* triple-negative breast cancer

### Knockdown of hsa_circ_001783 inhibits the progression of breast cancer cells

**Fig. 4:**
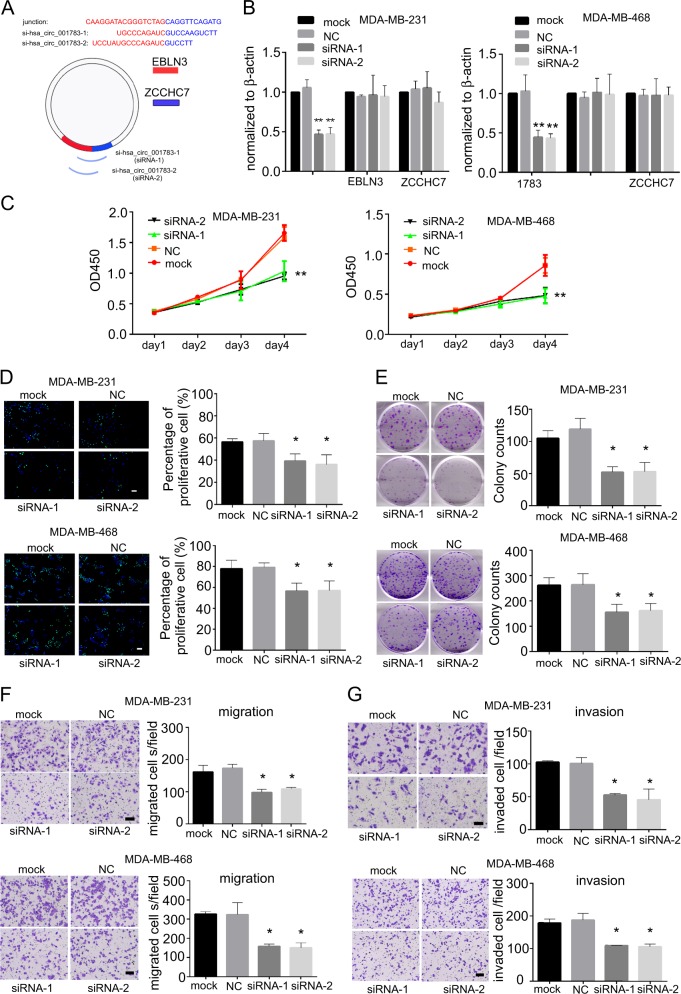
Effect of hsa_circ_001783 on MDA-MB-231 and MDA-MB-468 progression. **a** Schematic representation of the siRNA sequences specifically targeted the junction site of hsa_circ_001783. **b** qPCR analysis of the transfection efficacy and off-target effects of siRNA on EBLN3 and ZCCHC7 expression in MDA-MB-231 and MDA-MB-468 after 24 h transfection. **P < 0.01 compared to mock; *P < 0.05 compared to mock. **c** The proliferation status of MDA-MB-231 and MDA-MB-468 determined by CCK-8 assay after hsa_circ_001783 knockdown. OD optical density. **P < 0.01 compared to mock. **d** EdU assay to determine DNA synthesis of MDA-MB-231 and MDA-MB-468 transfected with siRNAs. All the data are shown as the mean ± SD; *P < 0.05 compared to mock. Scale bar, 100 μm. **e** Colony formation ability of MDA-MB-231 and MDA-MB-468 transfected with siRNA. All the data are shown as the mean ± SD; *P < 0.05 compared to mock. **f** The representative images of migrated MDA-MB-231 and MDA-MB-468 after hsa_circ_001783 knockdown. All the data are shown as the mean ± SD; *P < 0.05, compared to mock. Scale bar, 100 μm. **g** The representative images of invaded MDA-MB-231 and MDA-MB-468 after hsa_circ_001783 knockdown. All the data are shown as the mean ± SD; *P < 0.05, compared to mock. Scale bar, 100 μm

Our clinical data showed the correlations between hsa_circ_001783 expression level and breast cancer progression, and the GO analysis indicated hsa_circ_001783-correlated genes played profound roles in malignancy. We thus fine-tuned the expression of hsa_circ_001783 and monitored its cellular effects on two TNBC cell lines (MDA-MB-231 and MDA-MB-468) that highly expressed hsa_circ_001783 (Fig. 3a). Transfection of siRNAs that specifically targeted junction site of hsa_circ_001783 reduced its expression by 40–50% without any effect on EBLN3 or ZCCHC7 (Fig. 4a, b). Knockdown of hsa_circ_001783 significantly inhibited cell variability of MDA-MB-231 and MDA-MB-468 in day 4 after siRNA transfection (Fig. 4c). In addition, the EdU assay showed that downregulation of hsa_circ_001783 impaired the proliferation of MDA-MB-231 and MDA-MB-468, which was in consistent with the data in CCK8 assay (Fig. 4d). Colony formation ability was also disrupted after hsa_circ_001783 knockdown (Fig. 4e).

Our clinical data showed that breast cancer patients with high expression of hsa_circ_001783 suffered from more metastases (Fig. 2f). As metastasis begins with primary cancer cells acquiring migration and invasion abilities^28,29^, we next analyzed the effects of hsa_circ_001783 on metastatic ability of breast cancer cells in vitro. We used Boyden chamber coated with or without matrigel to evaluate the invasion and migration ability of breast cancer cells, respectively. After knockdown of hsa_circ_001783, migration ability and invasion capacity of the MDA-MB-231 and MDA-MB-468 were significantly decreased (Figs. 4f, g). Of noted, the proliferative status of these two cell lines was not significantly altered at the migration or invasion time point, suggesting that hsa_circ_001783 played a role in regulating the metastasis of breast cancer cells.

### Identification of hsa_circ_001783-correlated miRNAs

**Fig. 5:**
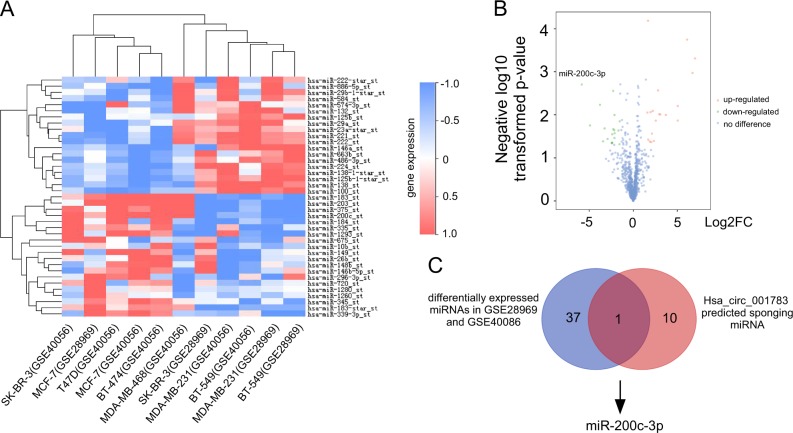
Validation of hsa_circ_001783-correlated miRNAs. **a** Heatmap of differentially expressed miRNAs with log_2_FC > 1.5 or log_2_FC < −1.5 in different breast cancer lines of two microarray datasets (GSE40056 and GSE28969). **b** Volcano plot of the differentially expressed miRNAs in GSE40056 and GSE28969 with log_2_FC > 1.5 or <−1.5 and *P* < 0.05. **c** Venn diagram showing the intersection between: blue circle, differentially expressed miRNAs between luminal/HER2+ breast cancer cell lines and triple-negative breast cancer cell lines; red circle, hsa_circ_001783 target miRNAs predicted by our analysis

Data mining of GEO datasets (GSE28969 and GSE40086) revealed that 19 miRNAs were higher and 20 miRNAs were lower in TNBC (MDA-MB-231, MDA-MB-468, and BT-549) than luminal/HER2+ cell lines (MCF-7, SK-BR-3, T47D, and BT-474; Fig. 5a, b; Supplementary Table 3). As hsa_circ_001783 expressed higher in TNBC than luminal/HER2+ cell lines (Fig. 3a), this suggests that these 19 miRNAs from GSE28969 and GSE40086 positively correlate with hsa_circ_001783 while other 20 miRNAs negatively correlate with hsa_circ_001783. Among these miRNAs, only miR-200c-3p was predicted as the target of hsa_circ_001783 (Fig. 5c).

### Hsa_circ_001783 contributes to breast cancer progression via sponging miR-200c-3p

**Fig. 6:**
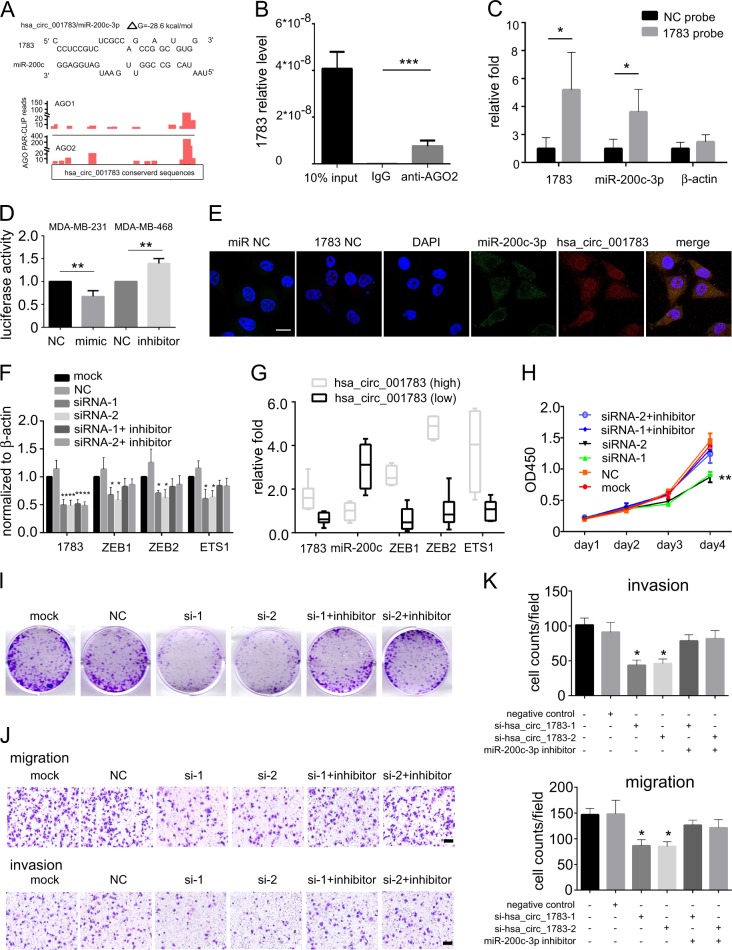
Hsa_circ_001783 serves as sponge for miR-200c-3p. **a** Examples of the potential bindings between hsa_circ_001783 conserved sequence and miR-200c-3p. Hsa_circ_001783 conserved locus is densely bound by AGO1 (red) and AGO2 (red). **b** AGO2 RNA-binding protein immunoprecipitation. 1783 is referred as hsa_circ_001783. All data are shown as the mean ± SD. ***P < 0.001 compared to IgG. **c** circRNA pull-down assay. 1783 is referred as hsa_circ_001783. **P < 0.01 compared to negative control (NC) probe. **d** Luciferase assay of MDA-MB-231 and MDA-MB-468 co-transfected with luciferase reporter containing hsa_circ_001783 conserved sequences and miR-200c-3p mimic or miR-200c-3p inhibitor. NC represents mimic negative control and inhibitor negative control. All data are shown as the mean ± SD. **P < 0.01 compared to negative control. **e** RNA fluorescence in situ hybridization for co-localization of hsa_circ_001783 and miR-200c-3p in MDA-MB-231. Scale bar 10 μm. **f** The expression of hsa_circ_001783 and miR-200c-3p mRNA targets: *ZEB1*, *ZEB2*, and *ETS1* after hsa_circ_001783 knockdown. **g** qPCR analysis of the expression of hsa_circ_001783, miR-200c-3p, and miR-200c-3p mRNA targets: *ZEB1*, *ZEB2*, and *ETS1* in breast cancer clinical specimens. **h** The proliferation status of MDA-MB-231 after siRNA transfection or miR-200c-3p inhibitor co-transfection determined by CCK-8. OD optical density. All data are shown as the mean ± SD; **P < 0.01 compared to mock. **i** Colony formation ability of MDA-MB-231 after siRNA transfection or miR-200c-3p inhibitor co-transfection. **j** The representative images of migrated and invaded MDA-MB-231 after siRNA transfection or miR-200c-3p inhibitor co-transfection. Scale bar, 100 μm. **k** The migration and invasion abilities of MDA-MB-231 after hsa_circ_001783 knockdown or miR-200c-3p inhibitor co-transfection. All the data are shown as the mean ± SD; *P < 0.05, compared to mock

To confirm the miR-200c-3p is the sponging target of hsa_circ_001783, we first calculated the number of potential binding sites between hsa_circ_001783 conserved sequences and miR-200c-3p by RNAhybrid. We found 18 bindings between them with the free energy <−20 kcal/mol (Supplementary Table 5). The one with the lowest free energy was shown in Fig. 6a. To further verify whether the miRNA bound to the conserved region of hsa_circ_001783, we analyzed three PAR-CLIP datasets of AGO1 and AGO2 (GSE28865, GSE43573, and GSE21918). The result showed a high density and degree of AGO1/2 occupancy within the conserved region of hsa_circ_001783 (Fig. 6a). To validate this result, we conducted AGO2 RIP and found that endogenous hsa_circ_001783 could be specifically pulled down by anti-AGO2 antibody (Fig. 6b). This suggests that hsa_circ_001783 acts as a miRNA-binding partner. By using probe targeting hsa_circ_001783 junction site, we found that hsa_circ_001783 and miR-200c-3p were significantly more abundant compared with the control (Fig. 6c). Moreover, according to a previously described method^24–26^ we conducted a luciferase assay by co-transfection of miR-200c-3p mimics and inhibitor with luciferase reporter into MDA-MB-231 and MDA-MB-468, respectively. Transfection of miR-200c-3p mimics reduced the luciferase reporter activity by 40%, while miR-200c-3p inhibitor promote the luciferase reporter activity by at least 1.4-fold (Fig. 6d). Of note, hsa_circ_001783 co-localized with miR-200c-3p in the cytoplasm of MDA-MB-231 cells (Fig. 6e). Besides, knockdown of hsa_circ_001783 enhanced miR-200c-3p expression and suppressed miR-200c-3p targeted genes: *ZEB1*, *ZEB2*, and *ETS1* (Fig. 6f; Supplementary Figure 1A). The negative correlation between hsa_circ_001783 and miR-200c-3p and positive correlation between hsa_circ_001783 and *ZEB1*, *ZEB2*, and *ETS1* were further observed in the tumor tissues from breast cancer patients (Fig. 6g). Transfection of miR-200c-3p inhibitor (miR-200c inhibitor) reduced the level of miR-200c-3p and enhanced migration and invasion of MDA-MB-231 (Supplementary Figure 1B). And co-transfection of hsa_circ_001783 siRNAs and miR-200c-3p inhibitor in MDA-MB-231 and MDA-MB-468 cells rescued cell proliferation and colony formation (Fig. 6h, i; Supplementary Figure 1C, D), migration and invasion (Fig. 6j, k; Supplementary Figure 1E, F). Together, these data suggest hsa_circ_001783 regulates breast cancer progression via sponging miR-200c-3p.

## Discussion

To the best of our knowledge, this is the first study that systematically analyzed the circular RNAs in breast cancer based on their characteristic in sponging disease specific miRNAs. By combination of our systematical pipeline, experimental technologies in vitro and ex vivo, we explored the role of circRNAs in progression of breast cancer via sponging miRNAs. Among all the breast cancer-associated circRNAs, hsa_circ_001783 was the one with the highest ranked score. Higher expression of hsa_circ_001783 associated with higher tumor burden and poorer prognosis of breast cancer. More importantly, hsa_circ_001783 regulated proliferation and metastasis of breast cancer cells via sponging miR-200c-3p.

Recently, circRNAs have been proved to act as miRNA sponges. Unlike other linear competitive endogenous RNA, circRNAs is more stable due to the covalently closed looped structure. The half-life of most circRNAs is longer than that of corresponding linear RNAs^4^. Previous studies focused on screening of the differentially expressed circRNAs rather than the circRNAs with sponging potential. However, if we merely screen circRNAs just based on the fold changes, it might result in ignoring some circRNAs which have more potent to sponge miRNAs. Besides, circRNAs is beyond miRNAs sponges as they exert their regulation role in coding peptides, interaction with proteins, and regulation on transcription^30,31^, which suggests screening differentially expressed circRNAs might lead to an uncertain direction.

Of note, we used five essential functional features of breast cancer to rank the circRNAs across the constructed circRNA–miRNA–breast cancer database. This strengthened the association between hsa_circ_001783 and breast cancer, although the total classes that hsa_circ_001783 sponged are not the best; for instance, hsa_circ_001851 harbors 13 classes of miRNA. Besides, we have uncovered that hsa_circ_001783 contributes to breast cancer progression via sponging miR-200c-3p and facilitates its prediction of clinical outcomes. Thus, the combination of high throughput computation, experimental technologies in vitro and clinical investigation turns out to be another efficient way to screen the “circular sponges” and potentially serves as a powerful approach to explore the novel predictors of clinical outcomes.

MiR-200c-3p has been reported to confer the progression abilities to breast cancer cells and exerts peculiar regulation roles in cancer proliferation, growth, migration, and invasion processes^32–34^. Interestingly, when we knocked down the expression of hsa_circ_001783, the proliferation, colony formation, and invasion abilities of breast cancer cells were suppressed, accompanied with the reduced expression of the miR-200c-3p targeted genes such as *ZEB1*/*2* (refs. ^32,33^) and *ETS1* (refs. ^34,35^), which are related to breast cancer proliferation and metastasis. Consistent with these in vitro findings, in clinical specimens, the expression level of *ZEB1*/*2* and *ETS1* is highly positively correlated with the expression of hsa_circ_001783. It is acknowledged that circRNAs sponge miRNAs and inhibit the function of miRNAs, which in turn up-regulates the mRNAs^11,13,16^. According to our data, we confirm that hsa_circ_001783 regulates *ZEB1*/*2* and *ETS1* via sponging miR-200c-3p, which is consistent with other published articles. For instance, after circHIPK3 knockdown, miR-124 is released and the miR-124 targeting genes such as *IL6R* and *DLX2* are down-regulated^13^. CDR1as can harbor miR-7 massively and *Fos*, *Klf4*, and *Nr4a3* targeted by miR-7 can be enriched in CDR1as knockout mice^11,16^. Therefore, our study indicates that hsa_circ_001783 can sponge miR-200c-3p and up-regulate miR-200c-3p targeting genes.

## Conclusion

In summary, we have proposed a comprehensive method to systematically screen breast cancer specific circular RNAs through integrating an in silico pipeline with in vitro and ex vivo techniques. We highlighted that hsa_circ_001783 as a novel prognostic marker for breast cancer and uncovered its new mechanism in regulating cancer proliferation and metastasis via sponging miR-200c-3p. Thus, this circular RNA  might be a potential therapeutic target for breast cancer treatment.

## Supplementary information

supplementary information

supplementary figure 1

supplementary table 1

supplementary table 2

supplementary table 3

supplementary table 4

supplementary table 5
