# Supplementary material for: Circular RNA hsa_circ_001783 regulates breast cancer progression via sponging miR-200c-3p
Source: Cell Death Dis. 2019 Jan 22;10(2):55. doi: 10.1038/s41419-018-1287-1 (PMC6343010; doi:10.1038/s41419-018-1287-1)
Supplement: Supplementary file 1 — supplementary information [file 41419_2018_1287_MOESM1_ESM.docx]

**Supplementary data**

Circular RNA hsa_circ_001783 regulates breast cancer progression via sponging miR-200c-3p

Zihao Liu^1#^, You Zhou^2#^, Gehao Liang^1^, Yun Ling^1^, Weige Tan^3^, Luyuan Tan^1^, Robert Andrews^2^, Wenjing Zhong^1^, Xuanxuan Zhang ^1^, Erwei Song^1,4^*, Chang Gong^1^*

1. Breast Tumor Center, Guangdong Provincial Key Laboratory of Malignant Tumor Epigenetics and Gene Regulation, Sun Yat-sen Memorial Hospital, Sun Yat-sen University, Guangzhou, China

2. Systems Immunity University Research Institute and Division of Infection and Immunity, School of Medicine, Cardiff University, Cardiff, United Kingdom

3. Department of Breast Surgery, First Affiliated Hospital of Guangzhou Medical University

4. Fountain-Valley Institute for Life Sciences，4th Floor, Building D Guangzhou Institute of Biomedicine and Health, Chinese Academy of Sciences, 190 Kaiyuan Avenue, Huangpu District Guangzhou China

^#^ Co-first author; *Correspondence

Correspondence: Chang Gong (gchang@mail.sysu.edu.cn) (changgong282@163.com) or Erwei Song (songerwei02@aliyun.com) (songew@mail.sysu.edu.cn)

**Inventory:**

Supplementary Figure legend

Supplementary Table legend

Supplementary Experimental Procedures

Supplementary Table legend

Supplementary Table 1. Prediction of potential bindings of miRNAs to the conserved sequences of individual circRNAs and association between circRNAs and different malignancy

Supplementary Table 2. Breast cancer associated circRNAs ranked by five essential features of breast cancer including: self-renewal/apoptosis, chemotherapy resistance, differentiation/proliferation, migration/invasion/metastasis and epithelia-mescenchymal-transition and circRNAs with the total scores≥ 21.

Supplementary Table 3. Differentially expressed genes between three TNBC cell lines (MDA-MB-231, MDA-MB-468 and BT-549) and luminal/HER2-overexpression cell lines in GSE41313 dataset with log_2_FC>1.5. Differentially expressed miRNAs between TNBC cell lines and luminal/HER2-overexpression cell lines of GSE40056 and GSE28969 datasets with log_2_FC>1.5

Supplementary Table 4. Correlation co-efficiency matrix of genes co-expressed with hsa_circ_001783 in MDA-MB-231, MDA-MB-468 and BT-549.

Supplementary Table 5. Potential bindings between hsa_circ_001783 conserved sequences and miR-200c-3p analyzed by RNAhybrid 2.2 with MEF<-20kcal/mol and primer sequences in this study.

Supplementary Figure legend

Supplementary Figure 1. Rescue experiments in MDA-MB-468. A. The level of miR-200c-3p after si-hsa_circ_001783 or miR-200c-3p inhibitor co-transfection. B. The level of miR-200c-3p after miR-200c-3p inhibitor transfection. The representative images of migrated and invaded MDA-MB-231 after miR-200c-3p inhibitor transfection. Scale bar, 100 μm. C. Colony formation ability of MDA-MB-468 after siRNA transfection and miR-200c-3p inhibitor co-transfection. D. The proliferation status of MDA-MB-468 after siRNA transfection and miR-200c-3p inhibitor co-transfection determined by CCK-8, OD, optical density. All data is shown as the mean ± SD.; **, *P*<0.01 compared to mock. E. The migration and invasion abilities of MDA-MB-468 after hsa_circ_001783 knock-down or miR-200c-3p inhibitor co-transfection. All data is shown as the mean ± SD.; * *p*<0.05, compared to mock. F. The representative images of migrated and invaded MDA-MB-468 after siRNA transfection or miR-200c-3p inhibitor co-transfection. Scale bar, 100 μm.

Supplementary experimental procedures

RNA isolation, RNASE R digestion and quantitative real-time PCR

Total RNA was extracted using TRIzol reagent (Life Technologies, USA) according to the manufacturer’s protocols. Total RNA was incubated with 2U RNASE R (Epicentre) at 37° C for 15 minutes and 85° C for 5 minutes. Complementary DNA was synthesized using the RT reagent kit (Vazyme, China) and the qPCR was performed using SYBR green reagent (Vazyme, China). For poly A tailed mRNA or non-poly A tailed RNA synthesis, random primer or oligo DT primer were used (Takara, Japan). MiR-200c-3p primer was purchased from GenePharma. The relative fold-change of gene expression was normalized to β-actin and was calculated by the 2-ΔΔCt method.

Primers and probes are listed as follow:

| Gene | Forward primer | Reverse primer |
| --- | --- | --- |
| hsa_circ_001783 | 5’-CGGGAGCTGTGACCCTTTC-3’ | 5’-AAAGACTGCTGCCCAGTGAAT-3’ |
| Beta-actin | 5’-TCATGAAGTGTGACGTGGACATC-3’ | 5’-CAGGAGGAGCAATGATCTTGATCT-3’ |
| GAPDH | 5’-AGGTGAAGGTCGGAGTCAAC-3’ | 5’-CGCTCCTGGAAGATGGTGAT-3’ |
| EBLN3 | 5’-CCTGGCTTTCCAAGGGCTAA-3’ | 5’-CGCTTCACTGACTGGCTTCC-3’ |
| Lnc-malat1 | 5’-AAAGCAAGGTCTCCCCACAAG-3’ | 5’-GGTCTGTGCTAGATCAAAAGGCA-3’ |
| ZCCHC7  ZEB1  ZEB2  ETS1  Sequence Primer | 5’-GTTATCCCAGAGCCATCC-3’  5’-CAGCTTGATACCTGTGAATGGG-3’  5’-GGAGACGAGTCCAGCTAGTGT-3’  5’-CCCTGGGTAAAGAATGCTTCC-3’  5’- CCGAGAGAGAGTCCAGTCTTTGAG-3’ | 5’-GCTTCTCCCAGTTTCTATTC-3’  5’-TATCTGTGGTCGTGTGGGACT-3’  5’-CCACTCCACCCTCCCTTATTTC-3’  5’-GCTGATGAAGTAATCCGAGGTG-3’  5’- CTCAAAAGACTGCTGCCCAGTG-3’ |
| MiR-200c-3p RT | 5’-CTCAACTGGTGTCGTGGAGTCGGCAATTCAGTTGAGTCCATCATTA-3’ | |
| MiR-200c qPCR | 5’-CTCAACTGGTGTCGTGGA-3’ | 5’-ACTGAAGTCGTGTGTAATACTGCC-3’ |
| hsa_circ_001783 probe | 5’-TTGGTTCCTATGCCCAGATCGTCCAAGTCTACTCGTTTTACA-3’ | |

Immunohistochemistry

Formalin-fixed breast cancer samples were deparaffinized with 100% xylene and rehydrated with different graded ethanol. After being treated with 3% H_2_O_2_, the specimen were treated with sodium citrate in 121° C to retrieve antigen and cool down to room temperature. After being incubated with 10% goat serum, the slides were treated with anti-Ki-67 antibodies (1:100 dilutions, CST, Cat log#9449) overnight and isotype-matched IgG was used as a negative control. The slides were washed with PBS for three times and incubated with HRP-linked secondary antibodies for one hour. Then standard HRP detection procedure was used to detect the slides. Cells with nuclear staining were regarded as positive cells. The percentage of Ki-67 positive cells in the whole fields of view was calculated to quantify the expression level of Ki-67 in each slide.

CCK8 assay

Cell proliferation state was assessed by CCK8 assay (Dojindo Laboratories, Japan). Cells (3 × 10^3^ per well) were seeded into 96-well plates and were incubated at 5% CO_2_ 37° C. CCK8 assay was repeated every day up to 4 days. For each assay, 10% of CCK8 reagent was added in each well and incubated for another 1 hour at 5% CO_2_, 37° C. Absorbance at 450nm was measured using microplate reader (Bio-Rad). Each independent experiment was repeated for three times.

EdU assay

The EdU assay was carried out using EdU assay kit (iClick™ EdU Andy Fluor 488 Imaging Kit, Genecopoeia, USA). Cells (5 × 10^3^) were seeded in 96-well plate after 24 hours transfection and incubated for 72 hours at 5% CO_2_ 37° C. After incubation with 0.5mM EdU for 24 hours, the cells were fixed with 4% formaldehyde and stained with Dye solution. The cells were washed with 3% BSA solution and Hoechst-33342 was carried out to stain nuclear. Images were acquired with fluorescence microscopy (Axio Observer D1, Germany).The EdU positive (EdU+) cells were regarded as the proliferative cells and percentage of proliferative cells was calculated as followed: the number of EdU+ cells divided by number of Hoechst33342+ cells. We calculated the positive cells in the whole fields of view. Independent experiments were repeated for three times.

Colony formation assay

After transfection, a total of 1000 cells were seeded in six-well plates and were incubated at 5% CO_2_ 37° C for 2 weeks. The colonies were fixed with 4% formaldehyde, stained with crystal violet and counted. Independent experiments were repeated for three times.

Boyden chamber assay

Invasion and migration capacities of breast cancer cells were examined using 24-well Boyden chamber with 8 µm pore filters (Corning, NY, USA). After transfection, 10^5^ cells per well breast cancer cells were suspended with serum-free medium and plated on the inserts in the upper chambers coated with or without of matrigel (Corning, NY, USA). After incubation for 9 or 24 hours for MDA-MB-231 and 12 or 24 hours for MDA-MB-468 at 5% CO_2_ 37° C, cells which did not invade or migrate were removed with cotton and cells that crossed the inserts were fixed with 4% formaldehyde, stained with crystal violet and were counted per field of view under microscope. The numbers of cells were counted in five random fields.

Nuclear-cytoplasmic fraction

Nuclear and cytoplasmic RNA was isolated with PARIS kit (Life Technologies, USA) following the manufacturer’s recommended procedure. Briefly, the cells were suspended and lysed with cell fraction buffer and then centrifuged at low speed to separate the nuclear fraction. Cell disruption buffer was added to the remained lysate which was later mixed with binding solution and equal volume of 100% ethanol. We drew the mixture and washed the samples for 2 times with washing solution. The RNA was eluted with elution solution.β-actin mRNA and long non coding RNA malat1, which only resided in nuclear, were referred as quality controls of nuclear and cytoplasm fractions respectively. Total RNA was the total portion of the nuclear part plus cytoplasm part and was referred to as the total RNA.
