# Supplementary figures and images for: Circular RNA hsa_circ_001783 regulates breast cancer progression via sponging miR-200c-3p
Source: Cell Death Dis. 2019 Jan 22;10(2):55. doi: 10.1038/s41419-018-1287-1 (PMC6343010; doi:10.1038/s41419-018-1287-1)

Supplementary Figure 1

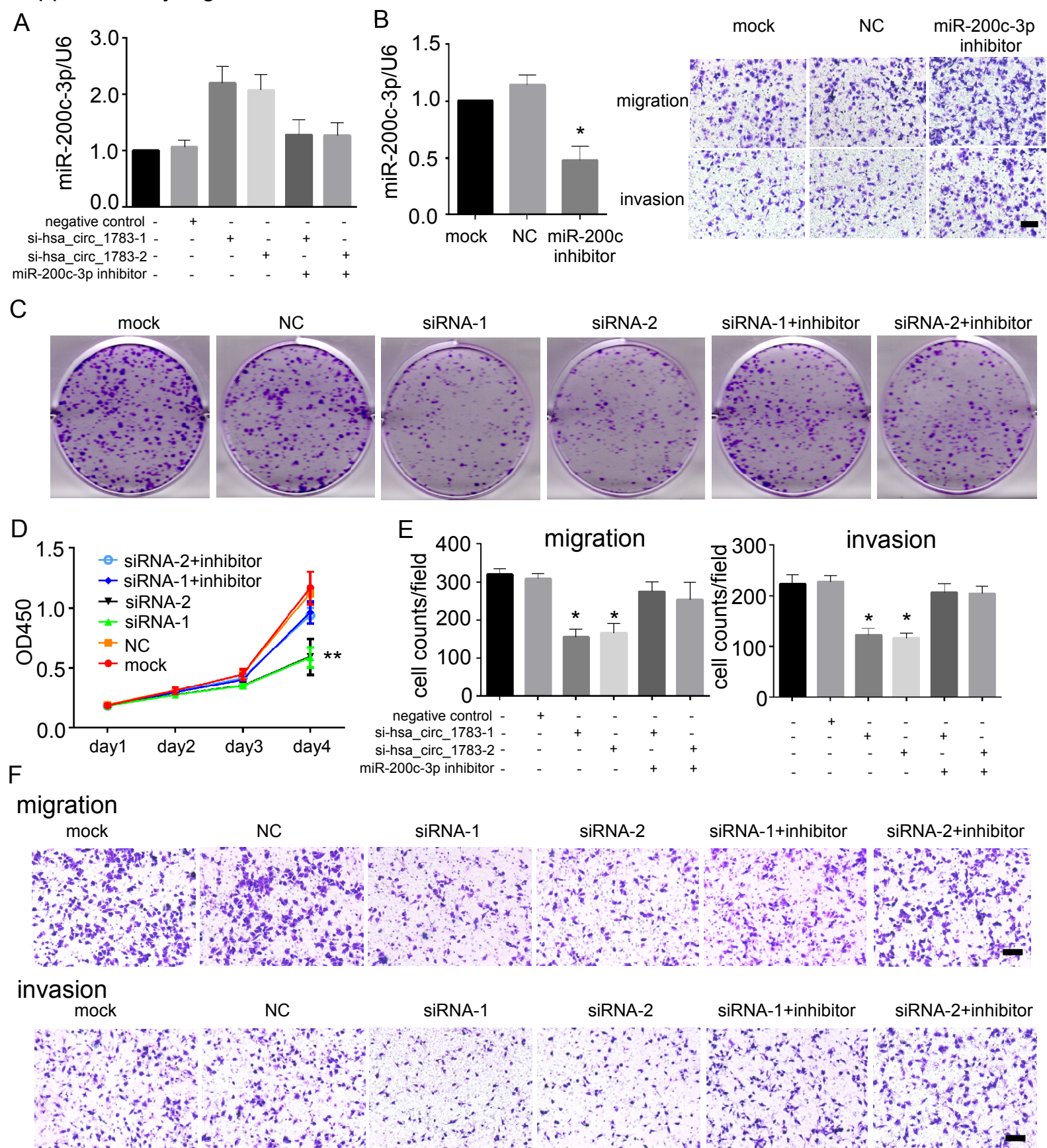

Supplement: Supplementary file 2 — supplementary figure 1 [file 41419_2018_1287_MOESM2_ESM.pdf]
