# Supplementary material for: Circular RNA hsa_circ_001783 regulates breast cancer progression via sponging miR-200c-3p
Source: Cell Death Dis. 2019 Jan 22;10(2):55. doi: 10.1038/s41419-018-1287-1 (PMC6343010; doi:10.1038/s41419-018-1287-1)
Supplement: Supplementary file 7 — supplementary table 5 [file 41419_2018_1287_MOESM7_ESM.docx]

Potential bindings between hsa_circ_001783 conserved sequences and miR-200c analyzed by RNAhybrid 2.2 with MEF<-20kcal/mol.

| position | Potential binding sites | mfe and p value |
| --- | --- | --- |
| 87 | target 5' G UGC AUUCACUG C 3'  CCUCUAUC AUUACC GGCAG  GGAGGUAG UAAUGG CCGUC  miRNA 3' G AUAAU 5' | mfe: -28.6 kcal/mol  p-value: 1.000000e+00 |
| 3258 | target 5' A A G 3'  UCC UCAU UACCCGGU  AGG AGUA AUGGGCCG  miRNA 3' GG U UCAUAAU 5' | mfe: -27.0 kcal/mol  p-value: 1.000000e+00 |
| 915 | target 5' U AA AGACUU C 3'  CCUUCA AUUACC GGCAG  GGAGGU UAAUGG CCGUC  miRNA 3' AG G AUAAU 5' | mfe: -26.9 kcal/mol  p-value: 1.000000e+00 |
| 1720 | target 5' U CG C G UU A 3'  CCUCC C UUA CU CAGUGU  GGAGG G AAU GG GUCAUA  miRNA 3' UA U G CC AU 5' | mfe: -24.6 kcal/mol  p-value: 1.000000e+00 |
| 994 | target 5' G UAA AA G 3'  CUCC UAU UACCUGGU  GAGG GUA AUGGGCCG  miRNA 3' G UA UCAUAAU 5' | mfe: -23.5 kcal/mol  p-value: 1.000000e+00 |
| 2427 | target 5' C CUU C G 3'  CCUUU UUAUUAUCC CAGUA  GGAGG AGUAAUGGG GUCAU  miRNA 3' U CC AAU 5' | mfe: -22.6 kcal/mol  p-value: 1.000000e+00 |
| 1628 | target 5' U UCU UUUCUUUUUUUUU UU G 3'  UCUCU UUGUU U GGCAGUG  GGAGG AGUAA G CCGUCAU  miRNA 3' U U GG AAU 5' | mfe: -22.1 kcal/mol  p-value: 1.000000e+00 |
| 751 | target 5' A AGAUGU UA A A 3'  CCAUCA UCU G GCAGUGU  GGUAGU GGG C CGUCAUA  miRNA 3' GGA AAU AU 5' | mfe: -22.1 kcal/mol  p-value: 1.000000e+00 |
| 582 | target 5' G A AA AG C A 3'  CUC GUUAU UGC GGC AGUG  GAG UAGUA AUG CCG UCAU  miRNA 3' G G GG AAU 5' | mfe: -21.8 kcal/mol  p-value: 1.000000e+00 |
| 152 | target 5' A A AGAUGAACCACAU A C 3'  UCCAUCG UGC CC GGCA  AGGUAGU AUG GG CCGU  miRNA 3' GG A CAUAAU 5' | mfe: -21.7 kcal/mol  p-value: 1.000000e+00 |
| 489 | target 5' A CG A C 3'  AUC UGCC GCAGU  UAG AUGG CGUCA  miRNA 3' GGAGG UA GC UAAU 5' | mfe: -21.7 kcal/mol  p-value: 1.000000e+00 |
| 1026 | target 5' A C CAGAUGGUUGC U C G 3'  CUUCCA UCAUU AUCC GC AG  GGAGGU AGUAA UGGG CG UC  miRNA 3' C AUAAU 5' | mfe: -21.6 kcal/mol  p-value: 1.000000e+00 |
| 3094 | target 5' A AGG GUUGA AUGGACAUAGGA UGACU G 3'  UUCCA UCA ACCC GGC GUGU  GAGGU AGU UGGG CCG CAUA  miRNA 3' G AA U AU 5' | mfe: -21.6 kcal/mol  p-value: 1.000000e+00 |
| 3433 | target 5' U GA U UAGUCUGAAUUAU U 3'  CUUCUA AUU CCUG GUAGUAUUG  GGAGGU UAA GGGC CGUCAUAAU  miRNA 3' AG U 5' | mfe: -21.4 kcal/mol  p-value: 1.000000e+00 |
| 2826 | target 5' G C G U G C G 3'  UCC UCA UAUCUG G GG AUUG  AGG AGU AUGGGC C UC UAAU  miRNA 3' GG U A G A 5' | mfe: -21.4 kcal/mol  p-value: 1.000000e+00 |
| 3489 | target 5' U U GUGAGUU U 3'  CCUCU UAUU CCU GGUAGU  GGAGG GUAA GGG CCGUCA  miRNA 3' UA U UAAU 5' | mfe: -21.3 kcal/mol  p-value: 1.000000e+00 |
| 884 | target 5' C G C UU UG A 3'  CCU UAUCA CCCG A GUUG  GGA GUAGU GGGC U UAAU  miRNA 3' G AAU CG CA 5' | mfe: -21.1 kcal/mol  p-value: 1.000000e+00 |
| 1982 | target 5' C CC UAAUUU A 3'  UCUCC UAUUACUU CAGUG  GGAGG GUAAUGGG GUCAU  miRNA 3' UA CC AAU 5' | mfe: -20.9 kcal/mol  p-value: 1.000000e+00 |
